# Dysfunction and ceRNA network of the tumor suppressor miR-637 in cancer development and prognosis

**DOI:** 10.1186/s40364-022-00419-8

**Published:** 2022-09-30

**Authors:** Jinze Shen, Chenhao Liang, Xinming Su, Qurui Wang, Yufei Ke, Jie Fang, Dayong Zhang, Shiwei Duan

**Affiliations:** Department of Clinical Medicine, Zhejiang University City College School of Medicine, Hangzhou, Zhejiang China

**Keywords:** miR-637, Cancer, ceRNA, Dysregulation, Prognosis, Drug resistance

## Abstract

MicroRNAs (miRNAs) are a class of small non-coding RNAs ranging from 17 to 25 nt in length. miR-637 is down-regulated in most cancers and up-regulated only in clear cell renal cell carcinoma (ccRCC). miR-637 can target 21 protein-coding genes, which are involved in the regulation of cell growth, cell cycle, cell proliferation, epithelial-mesenchymal transition (EMT), cancer cell invasion and metastasis, etc. In glioma, the transcription factor ZEB2 can bind to the miR-637 promoter region and inhibit miR-637 expression. Besides, miR-637 could be negatively regulated by competing endogenous RNA (ceRNAs) comprising 13 circular RNA (circRNAs) and 9 long non-coding RNA (lncRNAs). miR-637 is involved in regulating five signaling pathways, including the Jak/STAT3, Wnt/β-catenin, PI3K/AKT, and ERK signaling pathways. Low miR-637 expression was significantly associated with larger tumors and later tumor node metastasis (TNM) staging in cancer patients. Low miR-637 expression was also associated with poorer overall survival (OS) in cancer patients such as glioblastoma and low-grade gliomas (GBM/LGG), non-small cell lung cancer (NSCLC), hepatocellular carcinoma (HCC), and ovarian cancer (OV). Low expression of miR-637 increases the resistance of colorectal cancer (CRC) and human cholangiocarcinoma (CHOL) cancer cells to three anticancer chemotherapeutics (gemcitabine (dFdC), cisplatin (DDP), and oxaliplatin (OXA)). Our work summarizes the abnormal expression of miR-637 in various cancers, expounds on the ceRNA regulatory network and signaling pathway involved in miR-637, and summarizes the effect of its abnormal expression on the biological behavior of tumor cells. At the same time, the relationship between the expression levels of miR-637 and its related molecules and the prognosis and pathological characteristics of patients was further summarized. Finally, our work points out the insufficiency of miR-637 in current studies and is expected to provide potential clues for future miR-637-related studies.

## Facts

- 1. The expression of miR-637 is down-regulated in almost all cancers, And the expression of miR-637 is only up-regulated in ccRCC
- 2. miR-637 exerts tumor suppressor effect in cancer through ceRNA network.
- 3. Low expression of miR-637 is closely related to the poor prognosis of cancer.

## Open questions

- 1. Additional ceRNA networks for miR-637 in cancer need to be discovered.
- 2. What is the relationship between miR-637 and resistance to other anticancer drugs?
- 3. What is the link between the expression of miR-637 and its host gene DAPK3?
- 4. Why is the abnormal expression pattern and role of miR-637 different in ccRCC than in other cancers?

## Introduction

MicroRNA (miRNA) is a small single-stranded non-coding RNA (ncRNA) with a length of 17 ~ 25nt. It can usually bind to the 3'-untranslated region (3’-UTR) of messenger RNA (mRNA) and affect the mRNA stability or protein translation process, thereby down-regulating gene expression [1]. ceRNAs are a class of transcripts that can competitively regulate miRNAs at the post-transcriptional level and can participate in a wide range of biological processes through the ceRNA/miRNA/mRNA axis [2]. Deregulated miRNAs in cancers have the potential as biomarkers for cancer diagnosis [3]. Due to the high abundance and stability of miRNAs in body fluids, miRNAs can be used as liquid biopsy biomarkers in cancer patients [4].

miR-637 is located in the fifth intron of death associated protein kinase 3 (DAPK3) in the 19p13.3 region. DAPK3 belongs to the superfamily of calcium-dependent serine/threonine kinases and can regulate apoptosis and autophagy in various tumors [5]. miR-637 is underexpressed in most cancers. miR-637 can directly target 21 protein-coding genes, thereby regulating cell cycle, growth, proliferation, epithelial-mesenchymal transition (EMT), cancer cell invasion and metastasis, and other cell behaviors. miR-637 is competitively bound by ceRNA in cancer, and the low expression of miR-637 promotes the occurrence and development of cancer. The target genes of miR-637 are involved in various signaling pathways, which in turn affect the progression of cancer. miR-637 can increase the sensitivity of colorectal cancer (CRC) cancer cells to drugs such as gemcitabine (dFdC), gemcitabine (DDP), and oxaliplatin (OXA) [6].

Although miR-637 has been extensively studied in cancer, there is currently no systematic review of miR-637. Our work provides an overview of the aberrant expression of miR-637 in cancer and summarizes the role of miR-637 in acting as a tumor marker and inhibiting tumor progression.

### miR-637 and its host gene DAPK3

Death-associated protein kinases are a family of five Ser/Thr kinases with conserved catalytic domains that are closely related to cell death. DAPK3 regulates programmed cell death, including apoptosis and autophagy [7]. miR-637 is located in 19p13.3, the fifth intron of DAPK3.Since there is no study on the correlation between DAPK3 and miR-637, we have calculated the correlation of miR-637 and DAPK3 expression in lung cancer (GSE19804) and found that the miR-637 expression level was significantly correlated with DAPK3 (*r* = 0.56 and *p* < 0.001), indicating that miR-637 expression may depend on the host gene DAPK3.

### miR-637 is abnormally expressed in human cancers

As shown in Table 1, miR-637 expression was decreased in 18 cancers. Among them, the expression level of miR-637 in various cancer tissues was significantly lower than that in adjacent tissues, including glioblastoma and low-grade gliomas (GBM/LGG) [8–11], papillary thyroid carcinoma (PTC) [12, 13], non-small cell lung cancer (NSCLC) [14], gastric cancer (GC) [15–17], hepatocellular carcinoma (HCC) [18–21], CRC [22], pancreatic ductal adenocarcinoma (PDAC) [23], human cholangiocarcinoma (CHOL) [24], oral squamous cell carcinoma (OSCC) [25], prostate cancer (PCa) [26], ovarian cancer (OC) [27, 28], triple-negative breast cancer (TNBC) [29], cervical cancer (CCa) [30], osteosarcoma (SaOS) [31, 32], multiple myeloma (MM) [33], chronic myeloid leukemia (CML) [34]. In addition, the expression level of miR-637 was lower in the cell lines of various cancer cells than the corresponding normal cell lines, including PTC [12, 13], GC [17], HCC [18, 21], OSCC [35], PDAC [23], CHOL [36], TNBC [37], breast cancer (BRCA) [38], SaOS [31], etc. In the serum of CHOL patients, the expression level of miR-637 was lower than that of healthy people [39]. Notably, in the cancer tissues of clear cell renal cell carcinoma (ccRCC) patients, the expression level of miR-637 was higher compared with the adjacent tissues [40]. This may be due to the decreased expression of circHIPK3 in ccRCC tumors, which restores the expression of miR-637 [40].

**Table 1:** General downregulated expression of miR-637 in different cancers

Systems	Diseases	Expression	Level	Normal Group	Disease Group	Ref
Nervous	GBM/LGG	downregulated	tissue	normal brain tissues from 15 healthy people	27 snap-frozen glioma tissues from 27 glioma patients	[9]
		downregulated	tissue	adjacent normal brain tissues from 40 gliomas patients	glioma tissues from 40 gliomas patients	[8]
	GBM	downregulated	tissue	28 non-neoplastic brain tissue samples	primary GBM surgical specimens from 161 GBM patients	[11]
		downregulated	tissue	normal human astrocytes from healthy people	GBM tissues from 71 GBM patients	[10]
Endocrine	PTC	downregulated	cell	Nthy-ori 3–1	TPC-1, CGTH-W3, IHH-4, HTH83, and SW579	[12, 13]
		downregulated	tissue	adjacent normal tissues	PTC tissues from 54 PTC patients	[12]
		downregulated	tissue	homologous adjacent normal tissues	paraffin-embedded tumor tissues from PTC patients	[13]
Respiratory	NSCLC	downregulated	tissue	adjacent normal tissues	NSCLC tissues from 74 male and 49 female NSCLC patients	[14]
Digestive	GC	downregulated	tissue	adjacent normal tissues	GC tissues from 30 GC patients	[15]
		downregulated	tissue	adjacent normal tissues	GC tissues from 30 male and 28 female	[16]
		downregulated	cell	GES-1	BGC- 823, CRL-5822, SGC-7901, and AGS	[17]
		downregulated	tissue	adjacent normal tissues	GC tissues from 10 patients	
	HCC	downregulated	tissue	10 of non-neoplastic and non-cirrhotic liver tissue	HCC tissue from 15 patients	[19]
		downregulated	tissue	normal liver tissues	HCC specimens from 52 patients	[21]
		downregulated	cell	MIHA	HepG2, Hep3B, Bel7404, and Huh-7	
		downregulated	tissue	adjacent normal tissue samples	HCC tissue samples from 63 patients	[18]
		downregulated	cell	THLE-3	Huh7, HepG2, MHCC-97H, and Hep3B	
		downregulated	tissue	adjacent normal tissues	HCC tissues from 46 patients	[20]
	CRC	downregulated	tissue	adjacent normal tissues	CRC tissues from 50 CRC patients	[22]
	PDAC	downregulated	tissue	adjacent non-neoplastic tissues	PDAC tissues from 25 PDAC patients	[23]
		downregulated	cell	HPDE	SW1990, BxPC-3, and Capan-2	
	CHOL	downregulated	cell	H69	TFK-1, SNU-869, SSP-25, RBE, HuCCT1, and HuH28	[36]
		downregulated	serum	serum from healthy people	serum from 40 CHOL patients	[39]
		downregulated	tissue	adjacent tissues	CHOL tissues from 41 CHOL patients	[24]
	OSCC	downregulated	cell	NOK	OSCC-15, Tca8113, SCC-9, SCC-25, and HSC-2	[35]
		downregulated	tissue	adjacent normal tissues	OSCC tissurs from 51 OSCC patients	[25]
Urinary	PCa	downregulated	tissue	matched adjacent normal tissues	PCa tissues from 65 PCa patients	[26]
	ccRCC	upregulated	tissue	matched adjacent normal renal tissues	ccRCC tissues from 40 ccRCC patients	[40]
Reproductive	OC	downregulated	tissue	adjacent normal tissues	10 OC tissue from OC patients	[28]
		downregulated	tissue	adjacent non-neoplastic tissues from 30 OC patients	OC tissues from 30 OC patients	[27]
	TNBC	downregulated	tissue	para-cancerous tissues	TNBC tissues from 60 TNBC patients	[29]
		downregulated	cell	MCF-7	MDA-MB-231	[37]
	BRCA	downregulation	cell	MCF-10A	MCF-7 and T-47D	[38]
	CCa	downregulation	tissue	adjacent normal tissues	CCa tissues from 20 CCa patients	[30]
Bone	SaOS	downregulated	tissue	normal tissues from 10 SaOS patients	SaOS tissues from 10 SaOS patients	[31]
		downregulated	cell	SW1353	U2OS	
		downregulated	tissue	chondroma tissues	SaOS tissues from 12 SaOS patients	[32]
	MM	downregulated	tissue	bone marrow specimens from 21 healthy donors	bone marrow specimens from 36 MM patients	[33]
	CML	downregulated	plasma	plasma from 42 patients respond to IM	plasma from 66 patients non-respond to IM	[34]

*GBM* glioblastoma multiforme, *LGG* low-grade glioma, *PTC* papillary thyroid carcinoma, *NSCLC* non-small cell lung cancer, *GC* gastric cancer, *HCC* hepatocellular carcinoma, *CRC* colorectal cancer, *PDAC* pancreatic ductal adenocarcinoma, *CHOL* human cholangiocarcinoma, *OSCC* oral squamous cell carcinoma, *PCa* prostate cancer, *ccRCC* clear cell renal cell carcinoma, *OC* ovarian cancer, *TNBC* triple-negative breast cancer, *CCa* cervical cancer, *BRCA* breast cancer, *SaOS* osteosarcoma, *MM* multiple myeloma, *CML* chronic myeloid leukemia, *IM* Imatinib

### Inhibitory effect of transcription factor ZEB2 on miR-637 expression

ZEB2, an EMT-related transcription factor, is closely associated with poor prognosis and malignant phenotype of tumors [41]. In GBM/LGG, ZEB2 can directly bind to two ZEB2 binding sites (CACCT) in the promoter region of miR-637, thereby inhibiting miR-637 and promoting the malignant phenotype of glioma [42].

### The effect of miR-637 on cell behaviors

**Fig. 1:**
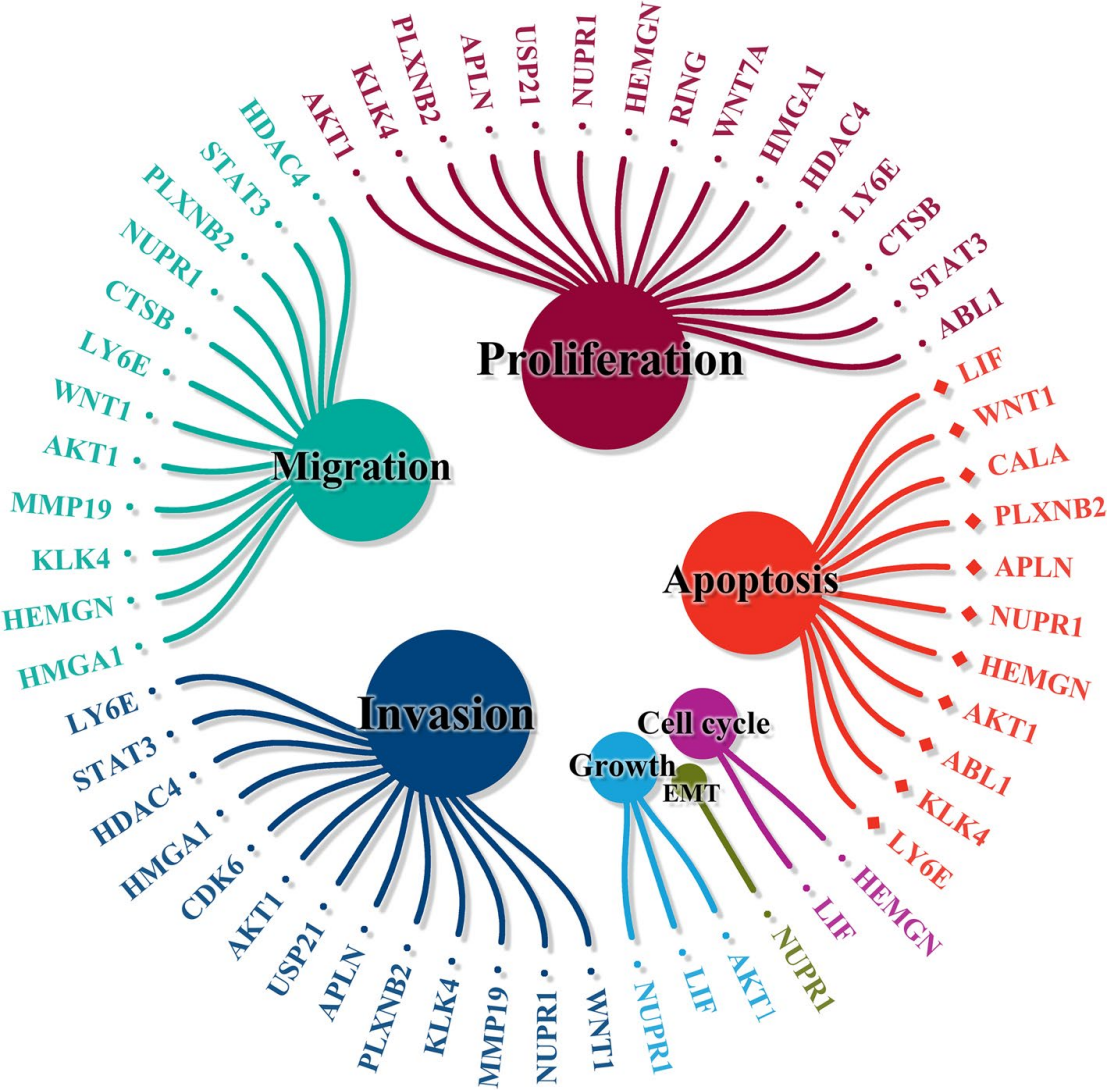
The target genes of miR-637 and cell behaviors. miR-637 inhibits cell cycle, proliferation, growth, EMT progression, invasion, and migration, and induces apoptosis by targeting the 3'-UTR of protein-coding genes.

Repression of protein-coding genes by miRNAs can also modulate various cancer cell behaviors [43]. As shown in Fig. 1 and Table 2, miR-637 can target and inhibit multiple genes, thereby regulating cell growth, cell cycle, cell proliferation, EMT, cancer cell invasion, and metastasis. There are 21 downstream target genes of miR-637, including 3 genes related to cell growth (AKT1, leukemia inhibitory factor (LIF), and NUPR1), 2 genes related to cell cycle (HEMGN and LIF), 15 genes related to cell proliferation, 11 genes related to apoptosis, 1 gene related to EMT (NUPR1), and 15 genes related to cancer cell invasion and metastasis.

**Table 2:** Roles of miR-637 in vivo and in vitro

Diseases	Targets	Effects in vitro	Cell lines	Effects in vivo	Laboratory animals	Ref
GBM/LGG	AKT1	growth↓, invasion↓, and migration↓	U251 and U87	tumorigenesis↓	nude mice	[9]
	HMGA1	proliferation↓, invasion↓, and migration↓	U251 and U87	tumor growth↓	nude mice	[42]
	CDK6	proliferation↓ and invasion↓	LN18	—	—	[50]
GBM	WNT7A	proliferation↓	U251 and LN229	tumor growth↓	BALB/c mice (4 weeks)	[10]
PTC	HEMGN	cell cycle↓, proliferation↓, migration↓, and apoptosis↑	TPC-1 and SW579	tumor growth↓	BALB/c mice (5 weeks)	[12]
	AKT1	proliferation↓, invasion↓, and migration↓	TPC-1 and HTH83	tumorigenesis↓	female BALB/C nude mice (8–12 weeks)	[13]
TC	KLK4	migration↓ and apoptosis↑	K1 and TPC-1	tumor growth↓	female BALB/C nude mice (5 weeks)	[52]
NSCLC	—	proliferation↓, invasion↓, and migration↓	H1299 and HCC827	—	—	[14]
GC	CALA	apoptosis↑	AGS	—	—	[55]
	APLN	proliferation↓, invasion↓, and apoptosis↑	SGC-7901	—	—	[15]
	MMP19	invasion↓ and migration↓	BGC823 and MGC803	—	—	[16]
	AKT1	invasion↓ and migration↓	SGC-7901 and AGS	—	—	[17]
HCC	LIF	growth↓ and apoptosis↑	HepG2 and Bel7404	tumor growth↓	female BALB/c nude mice (4–6 weeks)	[21]
	USP21	proliferation↓ and invasion↓	HepG2 and Hep3B	—	—	[51]
	AKT1	proliferation↓ and invasion↓	Huh-7 and Sk-Hep-1	—	—	[20]
	AKT1	proliferation↓ and invasion↓, migration↓	Huh7 and MHCC-97H	—	—	[18]
CRC	NUPR1	invasion↓ and migration↓	HCT116 and HT29	—	—	[22]
	WNT1	invasion↓, migration↓, and apoptosis↑	HCT116 and SW480	—	—	[56]
PDAC	AKT1	growth↓ and apoptosis↑	Capan-2 and BxPC-3	—	—	[23]
CHOL	LY6E	proliferation↓, invasion↓, migration↓, and apoptosis↑	HuCCT1 and RBE	tumor growth↓, metastasis ↓	nude mice	[36]
	CTSB	proliferation↓ and migration↓	QBC939	—	—	[24]
OSCC	NUPR1	proliferation↓	SCC-9 and HSC-2	—	—	[25]
	NUPR1	growth↓, invasion↓, EMT↓, and apoptosis↑	Tca8113 and SCC-9	tumor growth↓, EMT↓	BALB/c nude mice (4–6 weeks)	[35]
ccRCC	—	invasion↑ and migration↑	Caki1 and ACHN	—	—	[40]
OC	KLK4	proliferation↓, invasion↓, and migration↓	OVCAR-3 and H8910	—	—	[28]
	PLXNB2	proliferation↓, invasion↓, migration↓, and apoptosis↑	SKOV3 and CAOV3	tumor growth↓	BALB/c nude mice	[27]
TNBC	LIF	cell cycle↓, apoptosis↑, and autophagy↑	MDA-MB-231	tumor growth↓, metastasis ↓	female nude mice	[29]
	AKT1	proliferation↓ and migration↓	BT-549 and MDA-MD-231	tumorigenesis ↓, migration ↓, metastasis ↓	female BALB/c nude mice	[37]
	AKT3	—	—	tumor growth↓	female BALB/c nude mice	
CCa	RING1	proliferation↓	SiHa and C-4 I	tumor growth↓	nude mice	[30]
SaOS	STAT3	invasion↓ and migration↓	U2OS and SW1353	—	—	[31]
	HDAC4	proliferation↓, invasion↓, and migration↓	HOS and U2OS	—	—	[32]
MM	NUPR1	proliferation↓, apoptosis↑, and autophagy↓	U266 and RPMI8226	—	—	[33]
CML	ABL1	proliferation↓ and apoptosis↑	K562	—	—	[34]

*GBM* glioblastoma multiforme, *LGG* low-grade glioma, *PTC* papillary thyroid carcinoma, *TC* thyroid gland carcinoma, *NSCLC* non-small cell lung cancer, *GC* gastric cancer, *HCC* hepatocellular carcinoma, *CRC* colorectal cancer, *PDAC* pancreatic ductal adenocarcinoma, *CHOL* human cholangiocarcinoma, *OSCC* oral squamous cell carcinoma, *ccRCC* clear cell renal cell carcinoma, *OC* ovarian cancer, *TNBC* triple-negative breast cancer, *CCa* cervical cancer, *SaOS* osteosarcoma, *MM* multiple myeloma, *CML* chronic myeloid leukemia, *EMT* epithelial-mesenchymal transition

### The inhibitory effect of miR-637 on cancer cell growth

Oncogenic signaling pathways can enhance the metabolic level of cancer cells to meet the energy requirements for cancer cell growth [44]. miRNAs can affect the metabolism of cancer cells by inhibiting the AMPK signaling pathway [43]. For example, miR-3619-5p can inhibit fatty acid oxidation in cancer cells for energy [45]. miR-637 inhibits the growth of tumor cell lines and tumor growth in xenograft animal models by targeting AKT1 in glioma [9] and PDAC [23], LIF in HCC[21], and NUPR1 in OSCC [35]. miR-637 can inhibit cell growth by regulating metabolism, but the specific molecular mechanism remains to be confirmed by more studies.

### The blocking effect of miR-637 on cancer cell cycle

Inactivation of tumor suppressor genes relaxes cell cycle arrest, which in turn leads to genomic instability and ultimately promotes cancer development [46]. miR-637 blocks cancer cell cycle progression by targeting HEMGN and LIF.

HEMGN encodes a hematopoietic-specific nuclear protein of unknown function. Overexpression of HEMGN in bone marrow cells promotes cellular expansion [47]. In PTC, miR-637 blocked the cell cycle progression of PTC cell lines (TPC-1 and SW579) by targeting HEMGN and inhibiting the PI3K/Akt signaling pathway. Among them, miR-637 has the most obvious blocking effect on the G1/S transition phase of the cell cycle [12].

LIF is the most pleiotropic member of the IL-6 cytokine family. LIF participates in various pathways such as JAK/STAT, MAPK, and PI3K, and plays various roles in different types of cells, including stimulating or inhibiting cell proliferation, differentiation, and survival [48]. In TNBC, miR-637 can block the cell cycle progression of TNBC cell line MDA-MB-231 by inhibiting LIF expression. Specifically, miR-637 has the most obvious blocking effect in the G1 phase of the cell cycle, and miR-637 can also inhibit tumor growth and metastasis in nude mice [29].

### The effect of miR-637 on cancer cell proliferation

Cell proliferation is strictly regulated in the normal body. When the signaling pathway that inhibits proliferation is disturbed, it will lead to abnormal cell proliferation and cause cancer [49].

miR-637 inhibits cell proliferation of cancer cells by targeting 15 target genes (Fig. 1). These miR-637-targeted genes include HMGA1 [42], CDK6 [50], and WNT7A [10] in glioma, HEMGN in PTC [12], APLN in GC [15], USP21 in HCC [51], LY6E [36] and CTSB [24] in CHOL, NUPR1 in OSCC [25] and MM [33], HDAC4 in SaOS [32], ABL1 in CML [34], KLK4 in OC [28], PLXNB2 in OC [27], AKT1 in TNBC [37], PTC [13], and HCC [18, 20], AKT3 in TNBC [37], and RING1 in CCa [30].

miR-637 can also suppress the proliferation of NSCLC tumor cells, it is worth noting that the target gene of miR-637 in NSCLC has not been reported [14]. miR-637 can hinder the growth of various tumors in xenograft animals by inhibiting various genes. These xenograft animals include xenografted BALB/c mouse models of PTC (HEMGN [12], AKT1 [13]), TNBC (AKT1 [37]), OC (PLXNB2 [27]), thyroid gland carcinoma (TC) (KLK4 [52]), and xenografted nude mouse models of GBM (HMGA1 [42]) and CHOL (LY6E [36]).

### Promoting effect of miR-637 on cancer cell apoptosis

Apoptosis is programmed cell death that does not cause an inflammatory response and is an important form of cell death [53]. Apoptosis maintains the balance between cell death and cell survival, and aberrant apoptosis escape is an important feature of cancer cells [54].

miR-637 promotes cancer cell apoptosis by targeting 11 protein-coding genes (Fig. 1). miR-637 can promote the apoptosis of various cancer cells by inhibiting multiple genes. These apoptosis-related genes inhibited by miR-637 include HEMGN in PTC [12], KLK4 in TC [52], CALA [55] and APLN [15] in GC, LIF in HCC [21] and TNBC [29], WNT1 in CRC [56], AKT1 in PDAC [23], LY6E in CHOL [36], NUPR1 in OSCC [35] and MM [33], PLXNB2 in OC [27], and ABL1 in CML [34].

### Inhibitory effect of miR-637 on epithelial-mesenchymal transition (EMT)

The process of differentiation of epithelial cells into mesenchymal cells is called EMT [57]. EMT is the first step in the invasion-metastatic cascade, in which epithelial cells lose adhesion and polarity and acquire a strong migratory capacity similar to mesenchyme [58]. miRNA can affect the EMT process and are closely related to tumorigenesis, metastasis, and treatment resistance [59]. By targeting NUPR1 and inhibiting the conversion of E-cadherin to N-cadherin, miR-637 inhibited EMT progression in OSCC cell lines (Tca8113 and SCC-9) and OSCC xenograft BALB/c nude mouse model [35].

### Attenuating effect of miR-637 on cancer cell invasion and metastasis

Invasion of tumor cells into surrounding tissues and metastasis in blood vessels are important initial steps of tumor metastasis and are the main cause of high mortality in cancer [60]. miR-637 inhibits the invasion and metastasis of various cancer cells by targeting 15 target genes (Fig. 1). These genes include HMGA1 in glioma [42], AKT1 in glioma [9], PTC [13], GC [17], and HCC [18, 20], HEMGN in PTC [12], APLN [15] and MMP19 [16] in GC, WNT1 in CRC[56], NUPR1 in CRC [22] and OSCC [35], LY6E [36] and CTSB [24] in CHOL, KLK4 [28] and PLXNB2 [27] in OC, and HDAC4 [32] and STAT3 [31] in SaOS.

Furthermore, miR-637 inhibited cancer cell invasion in glioma [50] and HCC [51] by targeting CDK6 and USP21, respectively. miR-637 also inhibited cancer cell metastasis of TC by targeting KLK4 [52]. In NSCLC, miR-637 inhibited cancer cell invasion and metastasis [14]. miR-637 can inhibit tumor metastasis in the CHOL xenograft nude mouse by targeting LY6E [36].

Notably, downregulation of circHIPK3 in ccRCC alleviated its repressive effect on the expression level of miR-637, thereby attenuating invasion and metastasis in Caki1 and ACHN cell lines [40].

### miR-637 is involved in various cancer-related signaling pathways

**Fig. 2:**
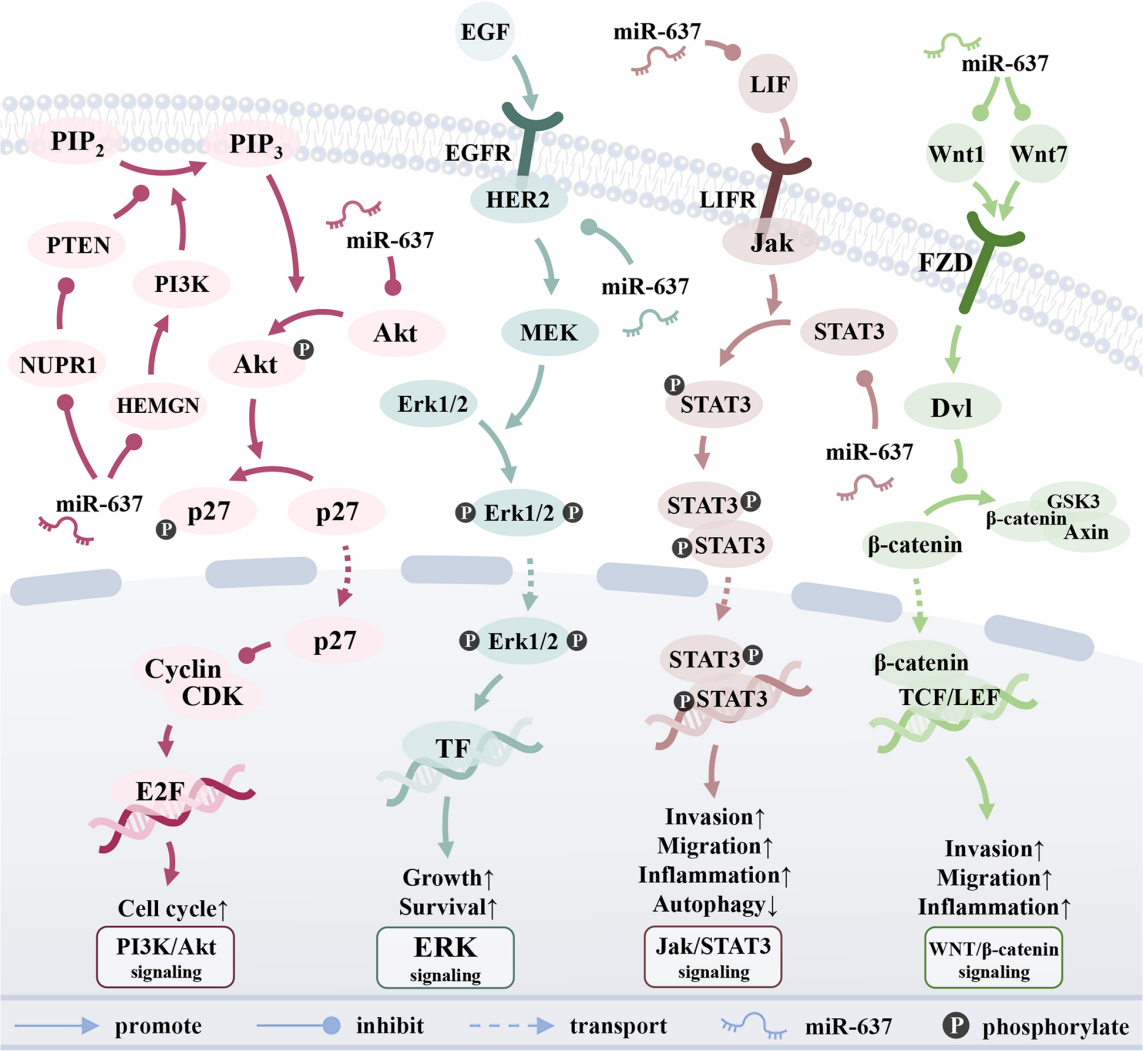
miR-637 is involved in the regulation of multiple signaling pathways. miR-637 plays an important role in Jak/STAT3 signaling pathway, ERK signaling pathway, Wnt/β-catenin signaling pathway, and PI3K/AKT signaling pathway, thereby regulating cell growth, apoptosis, cell cycle, invasion, and metastasis.

As shown in Fig. 2, miR-637 can regulate four signaling pathways, thereby inhibiting the occurrence and development of cancer. The signaling pathways associated with miR-637 in cancer include the Jak/STAT3 signaling pathway [26], the Wnt/β-catenin signaling pathway [56], the ERK signaling pathway [61], and the PI3K/AKT signaling pathway [12].

### The Jak/STAT3 signaling pathway

In the tumor microenvironment, the Jak/STAT3 signaling pathway drives tumor cell proliferation, survival, invasion, and metastasis, and suppresses anti-tumor-related immune responses [62]. lncAMPC can bind to histone H1.2 and promote the transcription of LIFR [26]. LIF activates Jak upon its binding to the LIFR receptor on the extracellular plasma membrane, thereby promoting the phosphorylation and dimerization of STAT3 into the nucleus, activating the signaling pathway [26]. Meanwhile, in CRC, low expression of miR-637 leads to activation of the Jak/STAT3 signaling pathway through the lncAMPC/miR-637/LIF axis, thereby promoting inflammation and cancer cell migration and invasion [6, 26].

### The Wnt/β-catenin signaling pathway

The WNT/ß-catenin signaling pathway regulates embryonic development, cell proliferation, and differentiation [63]. Wnt1 and Wnt7, encoded by miR-637 targets WNT1 and WNT7A, can coil Frizzled (FZDs) family receptors and recruit Dvl1 to activate the Wnt/β-catenin pathway [63]. In cancer, low levels of miR-637 lead to up-regulation of Wnt1 in CRC [56] and Wnt7 in GBM [10] and activate the Wnt/β-catenin pathway, thereby promoting cancer cell invasion, metastasis, metabolism, and inflammation [64].

### The ERK signaling pathway

The ERK pathway is associated with cell proliferation, differentiation, migration, senescence, and apoptosis [65]. The polypeptide growth factor EGF activates the membrane tyrosine kinase HER2 by binding to the receptor EGFR on the plasma membrane, promoting MEK phosphorylation and Erk1/2 diphosphorylation, thereby activating the ERK signaling pathway [61]. In HER2-positive BRCA, miR-637 can down-regulate HER2, inhibit MEK phosphorylation and Erk signaling pathway, and ultimately promote cell apoptosis and inhibit proliferation and differentiation [61].

### The PI3K/AKT signaling pathway

Aberrant activation of the PI3K/AKT pathway in cancer leads to cellular competitive growth advantage, metastatic capacity, angiogenesis, and therapeutic drug resistance [66]. HEMGN can recruit and activate AKT on the plasma membrane by promoting the phosphorylation of PI3K and the generation of PIP3, resulting in the activation of the PI3K/AKT signaling pathway [12]. NUPR1 can inhibit PTEN, a negative regulator of AKT, and phosphorylate PIP3 [67], which in turn promotes AKT phosphorylation and activates the PI3K/AKT signaling pathway [68]. Low expression of miR-637 leads to up-regulation of the expression levels of HEMGN in PTC [12], NUPR1 in OSCC [35], and AKT1 in glioma [9, 69] and PDAC [23], thereby activating the PI3K/AKT signaling pathway and inhibiting the reproduction and growth of cancer cells.

### The miR-637-related ceRNAs

CircRNA is a class of endogenous non-coding RNAs with a covalently closed-loop structure, without a 5'-cap and a 3'-poly-A tail, and plays an important role in the occurrence and development of human diseases, especially tumors [70]. LncRNAs have been proved to be the main regulators of gene expression in recent years, and play an important regulatory role in the occurrence and development of various diseases including cancer [71]. CircRNA and long non-coding RNA (lncRNA) increase the expression level of miRNA target genes by competitively binding to miRNA. The ceRNA network can link protein-coding mRNAs with miRNAs, lncRNAs, circular RNA (circRNAs), and other non-coding RNAs [72].

**Fig. 3:**
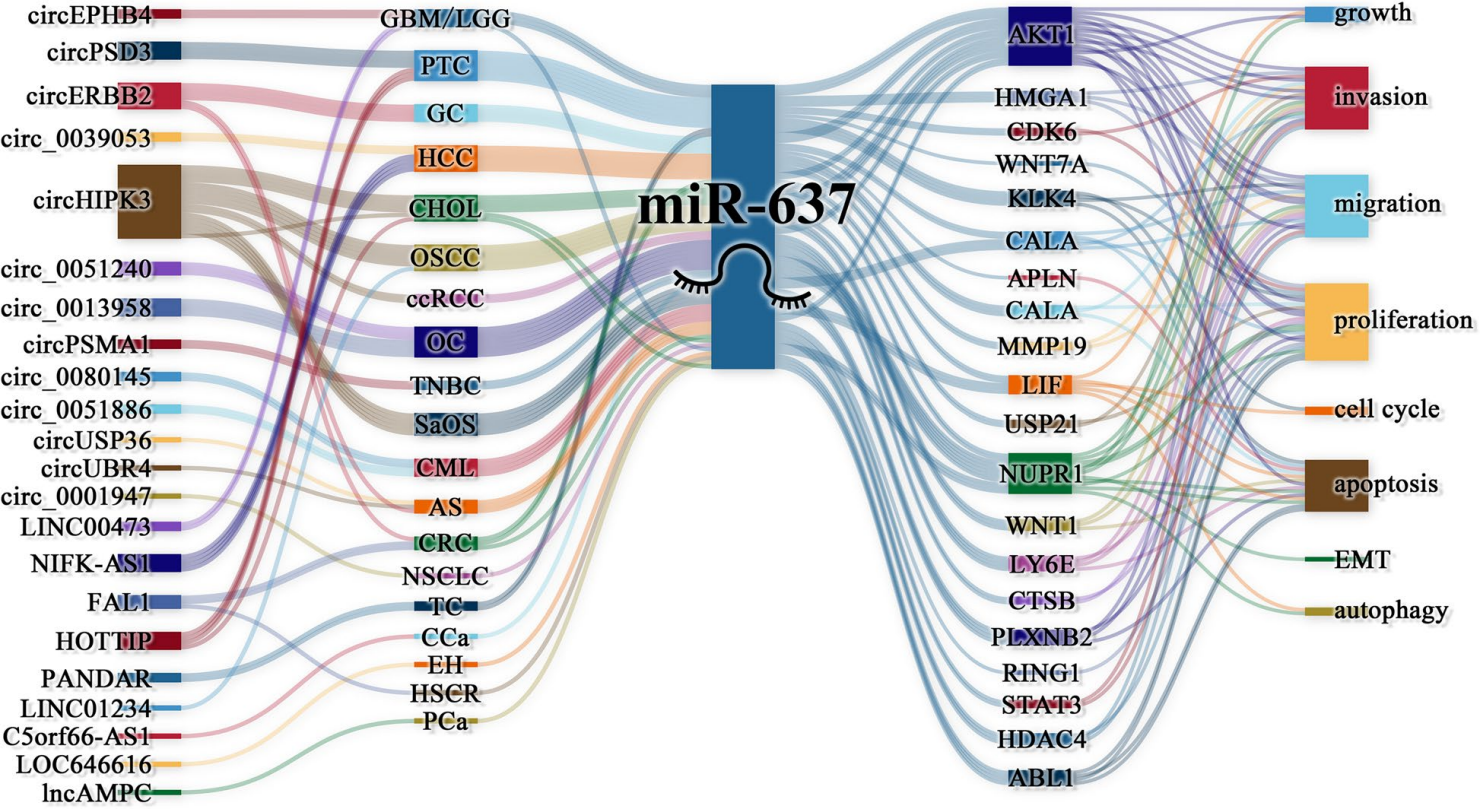
The ceRNA networks of miR-637. miR-637 is involved in various ceRNA/miRNA regulatory axes and regulates various cellular biological behaviors including cell cycle, proliferation, apoptosis, invasion, and metastasis.

As shown in Table 3 and Fig. 3, the miR-637-related ceRNA regulatory network is associated with the occurrence and development of various cancers. In cancer and other diseases, various circRNAs can inhibit the expression of miR-637. These circRNAs include circHIPK3 (CRC [6], CHOL [73], and atherosclerosis [74]), circEPHB4(GBM/LGG) [8], circ_0001947(NSCLC) [75], circ_0080145(CML) [34], circ_0051886(CML) [34], circUSP36(atherosclerosis) [76], and circUBR4(atherosclerosis) [77].

**Table 3:** The binding sites of ceRNAs on miR-637

ceRNA axes	Diseases	Binding site of ceRNA and miR-637		Binding site of miR-637 and PCG		Ref
		Binding sites of ceRNA (5'-…-3')	Binding sites of miR-637(3'-…-5')	Binding sites of PCG (3'-…-5')	Binding sites of miR-637 (5'-…-3')	
circ_0001947/miR-637	NSCLC	—	—	—	—	[75]
circ_0013958/miR-637/PLXNB2	OC	—	—	GACCCCC	CUGGGGG	[27]
circ_0051240/miR-637		CCCCCAG	GGGGGUC	—	—	[28]
circ_0039053/miR-637	HCC	CAAGtCtctcAtCCCCCAG	GUcUCgGgcuuUcGGGGGUC	—	—	[51]
circ_0051886/miR-637/ABL1	CML	GACCCC	CUGGGG	CCCCAG	GGGGUC	[34]
circEPHB4/miR-637/SOX10	GBM/LGG	CCCGccuCCCCCAG	GGGCuucGGGGGUC	ACCCCCGggAcCCCGgGgCG	UGGGGGCuuUcGGGCuCuGC	[8]
circERBB2/miR-637	GC	—	—	—	—	[16]
circHIPK3/miR-637/NUPR1	OSCC	GCAtttCCtaGAAgGCCCCCAG	CGUcucGGgCUUuCGGGGGUC	GACCCCC	CUGGGGG	[35]
circHIPK3/miR-637/LY6E	CHOL	—	—	CCCCCAG	GGGGGUC	[36]
circHIPK3/miR-637/STAT3	CRC	GCAtttCCtaGAAgGCCCCCAG	CGUcucGGgCUUuCGGGGGUC	GACCCCgcAAA	CUGGGGcgUUU	[6]
circPSD3/miR-637	PTC	CCCCAG	GGGGUC	—	—	[12]
circPSMA1/miR-637	TNBC	AGCCCCCAG	UCGGGGGUC	—	—	[37]
circUBR4/miR-637/FOXO4	AS	CCCCCAG	GGGGGUC	GACCCCC	CUGGGGG	[77]
circUSP36/miR-637/WNT4		CCCCCAG	GGGGGUC	GACCCC	CUGGGG	[76]
C5orf66-AS1/miR-637/RING1	CCa	GCCCCCAG	CGGGGGUC	UGACCCCC	ACUGGGGG	[30]
FAL1/miR-637/AKT1	HSCR	GCCtGggtcgCCCCAG	CGGgCuuucgGGGGUC	GACCCCC	CUGGGGG	[78]
HOTTIP/miR-637/LASP1	CHOL	GCCCCCAGU	CGGGGGUCA	TGACCCCC	ACUGGGGG	[39]
HOTTIP/miR-637	PTC	GuAaAuauuGCCCCCAGU	CgUcUcgggCUuuCGGGGGUCA	—	—	[13]
lncAMPC/miR-637	PCa	GtAtttAcAtCCCCCAGT	CgTgggTtTcGGGGGTCA	—	—	[26]
LINC00473/miR-637/CDK6	GBM/LGG	GCAGCCCGAgACCCCCAG	CGUCGGGCUuUGGGGGUC	GACCCCC	CUGGGGG	[50]
LINC01234/miR-637/NUPR1	OSCC	CCCCCAGU	GGGGGUCA	GACCCCC	CUGGGGG	[25]
LOC646616/miR-637	EH	AGAauCCaAgcaCCCCCAG	UCUcgGGcUuucGGGGGUC	—	—	[79]
NIFK-AS1/miR-637	HCC	CCCCAGA	GGGGUCU	—	—	[18]
PANDAR/miR-637/KLK4	TC	AAAACAG	GGGGGUC	GACCCCCG	CUGGGGGC	[52]

*PCG* protein-coding gene, *NSCLC* non-small cell lung cancer, *OC* ovarian cancer, *EH* essential hypertension, *HCC* hepatocellular carcinoma, *CML* chronic myeloid leukemia, *GBM* glioblastoma multiforme, *LGG* low-grade glioma, *GC* gastric cancer, *OSCC* oral squamous cell carcinoma, *PTC* papillary thyroid carcinoma, *TNBC* triple-negative breast cancer, *AS* atherosclerosis, *CCa* cervical cancer, *HSCR* Hirschsprung’s disease, *CHOL* human cholangiocarcinoma, *PCa* prostate cancer, *TC* thyroid gland carcinoma

CircRNAs and lncRNAs act as ceRNAs of miR-637 on its target mRNAs and play important roles in various biological processes of cancer. The circPSMA1/miR-637/AKT1 axis in TNBC enhances the proliferation and migration ability of TNBC cell lines and promotes tumor metastasis in BALB/c mice [37]. The circERBB2/miR-637/MMP19 axis in GC enhances the invasive and migratory abilities of GC cell lines [16]. In OC, the circ_0051240/miR-637/KLK4 [28] axis and the circ_0013958/miR-637/PLXNB2 axis [27], promote the malignant phenotype of OC cell lines. The circ_0039053/miR-637/USP21 axis in HCC promotes the proliferation and migration ability of HCC cell lines [51]. In PTC, the circPSD3/miR-637/HEMGN axis promotes the proliferation, migration, and cell cycle progression of PTC cell lines, and inhibits apoptosis [12]; in addition, the circHIPK3/miR-637/AKT1 axis in GC [17], the circHIPK3/miR-637/NUPR1 axis in OSCC [35], and the circHIPK3/miR-637/STAT3 axis in SaOS [31] are associated with cancer progression. In CRC, the lncFAL1/miR-637/NUPR1 axis promotes the invasion and migration of CRC cell lines [22, 78]. In HCC, the NIFK‑AS1/miR-637/AKT1 axis enhanced the proliferation, invasion, and migration of HCC cell lines [18]. In OSCC, the LINC01234/miR-637/NUPR1 axis promotes the proliferation of OSCC cell lines [25]. In CCa, the C5orf66-AS1/miR-637/RING1 axis enhances the proliferative capacity of CCa cell lines and tumor growth in nude mice [30]. In TC, the HOTTIP/miR-637/AKT1 axis [13] and the PANDAR/miR-637/KLK4 axis [52], promote the malignant phenotype of TC cell lines. In glioma, the LINC00473/miR-637/CDK6 axis promotes the proliferation and invasion of glioma cell lines [50]. The lncAMPC/miR-637/LIF axis in PCa [26], HOTTIP/miR-637/LASP1 axis in CHOL [39], LOC646616/miR-637 axis in essential hypertension [79], exert oncogenic effects in cancer. Notably, only in ccRCC, circHIPK3 expression was decreased, leading to up-regulation of miR-637 levels, which promoted the invasion and migration of Caki1 and ACHN cell lines [40].

### Prognostic value of miR-637

The dysregulated expression of miR-637 is closely related to the clinicopathological features of cancer. As shown in Table 4, existing data confirmed that in GBM/LGG, NSCLC, HCC, and ovarian cancer (OV).

**Table 4:** Prognostic values of miR-637 and miR-637 related genes

Diseases	Sample size	prognostic factor	Relationship with miR-637	Expression in high-risk patients	Clinicopathological characteristic	Prognostic value	Ref
GBM/LGG	70	miR-637	—	Low	associated with clinical stage	OS	[9]
GBM	161	miR-637	—	Low	—	OS	[11]
NSCLC	123	miR-637	—	Low	associated with TNM stage	OS	[14]
OV	281	miR-637	—	Low	—	OS	[80]
HCC	61	miR-637	—	Low	—	OS	[51]
	61	circ_0039053	Negative correlation	High	associated with TNM stage and lymph node metastasis	OS	[51]
	96	NIFKAS1	Negative correlation	High	—	OS and DFS	[18]
CRC	179	circHIPK3	Negative correlation	High	associated with recurrence, tumor size, regional lymph node metastasis, and distant metastasis	OS and DFS	[6]
TNBC	60	circSEPT9	Negative correlation	High	associated with TNM stage	OS	[29]
PCa	237	LIF	Negative correlation	High	associated with tumor metastasis	RFS	[26]

*GBM* glioblastoma multiforme, *LGG* low-grade glioma, *NSCLC* non-small cell lung cancer, *HCC* hepatocellular carcinoma, *OV* ovarian cancer, *CRC* colorectal cancer, *TNBC* triple-negative breast cancer, *PCa* prostate cancer, *TNM* tumor node metastasis, *OS* overall survival, *DFS* disease-free survival, *RFS* relapse-free survival

, low expression of miR-637 was closely associated with poor patient prognosis, suggesting that miR-637 could be used as a biomarker for cancer prognosis. In GBM/LGG, low expression levels of miR-637 and high expression of AKT1 generally represent tumor progression and are associated with poorer overall survival (OS) and higher clinical stage [9]. In GBM, low expression of miR-637 and high expression of its target CYBRD1 were associated with poorer OS [11]. In NSCLC, lower levels of miR-637 were associated with lower OS and later tumor node metastasis (TNM) stage [14]. In HCC and OV, low levels of miR-637 predict lower OS in patients [51, 80].

Besides, the ceRNA networks of miR-637 also have a high clinical value. In HCC, circ_0039053/miR-637/USP21 axis usually predicts a higher TNM stage, lymph node metastasis rate, and lower OS in patients [51]. NIFKAS1/miR-637/Akt1 axis generally indicates lower OS and disease-free survival (DFS) in HCC patients [18]. In CRC, circHIPK3/miR-637/STAT3 axis is associated with lower OS and DFS, larger tumor volume, higher probability of regional lymph node metastasis, distant metastases, and poor recovery [6]. In TNBC, circSEPT9/miR-637/LIF/STAT3 axis is associated with a higher TNM stage and lower OS in patients [29]. In PCa, lncAMPC/LIF/LIFR axis predicts a higher tumor metastasis rate and lower relapse-free survival (RFS) in patients [26].

### miR-637 and drug resistance in cancer cells

**Fig. 4:**
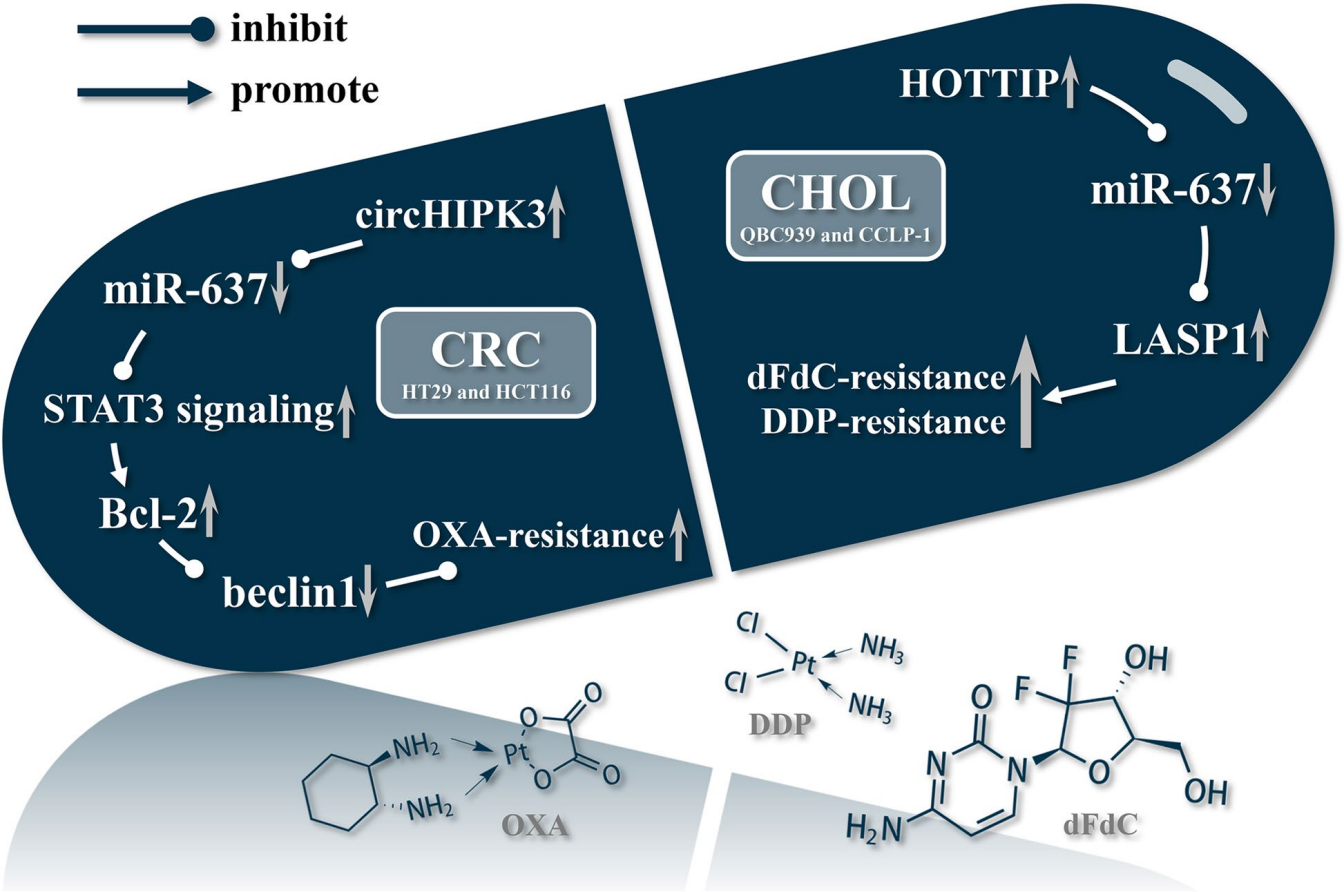
The roles of miR-637 in the development of drug resistance. The low expression of miR-637 leads to the resistance of cancer cells to chemotherapeutic drugs, including OXA, dFdC, and DDP, by affecting the levels of a series of downstream proteins.

As shown in Fig. 4, abnormally low expression of miR-637 was associated with the resistance of cancer cells to three anticancer drugs dFdC, cisplatin (DDP), and OXA.

dFdC is the most important low molecular weight cytidine analog since cytarabine (Ara-C) [81]. DDP is a neutral square planar coordination complex of platinum (II), which is widely used in the treatment of various cancers [82]. dFdC-DDP is currently the standard therapy for advanced biliary tract cancer, but the generation of drug resistance is still an important problem in clinical treatment [83, 84]. LASP1 is a multifunctional protein that plays an important role in cytoskeleton formation [85]. LASP1 inhibits the sensitivity of cancer cells to chemicals [86]. In CHOL, miR-637 significantly inhibited the expression level of LASP1, which in turn enhanced the sensitivity of QBC939 and CCLP-1 cells to dFdC and DDP [39]. The level of miR-637 was competitively inhibited by its molecular sponge lncHOTTIP, resulting in the resistance of cancer cells to dFdC and DDP [39].

OXA is a third-generation platinum-based anticancer drug, mainly used for the treatment of CRC [87]. miR-637 can inhibit the expression of STAT3, thereby inhibiting the activation of the STAT3 signaling pathway, resulting in a decrease in the level of downstream Bcl-2, an increase in the level of beclin1, and enhancement of autophagy. The level of miR-637 was competitively inhibited by its molecular sponge circHIPK3, resulting in the resistance of HT29 and HCT116 cells to OXA [6].

## Discussions

Various ceRNAs are aberrantly expressed in cancer, and multiple studies have exploited the interrelationships among dysregulated lncRNAs, circRNAs, miRNAs, and mRNAs to construct cancer-associated ceRNA networks [88]. The study of the ceRNA network helps to predict pathological changes in cancer patients and provides new molecular markers for prognosis [89, 90].

miR-637 is a potential cancer biomarker with diagnostic and prognostic value. The expression level of miR-637 in cancer cells or tissues was generally lower than that in corresponding normal cells or tissues, and the expression level of miR-637 was only up-regulated in ccRCC. Low expression of miR-637 is closely associated with poor prognosis in GBM/LGG, NSCLC, HCC, and OV cancer patients. An increasing number of studies have shown that miR-637 exerts tumor suppressor effects through multiple pathways in most cancers (except ccRCC). miR-637 can directly target the 3'-UTR of 21 target genes, participate in at least 5 signaling pathways (Jak/STAT3, Wnt/β-catenin, PI3K/AKT, and ERK), and regulate the complex ceRNA axis and related network. miR-637 blocks the cell cycle in most cancers (except ccRCC), inhibits cancer cell growth, proliferation, EMT, invasion, and metastasis, and inhibits tumorigenesis and progression. Meanwhile, miR-637 also played a regulatory role in the resistance of cancer cells to three anticancer drugs (dFdC, DDP, and OXA).

The host gene of miR-637, DAPK3, is a nuclear protein kinase involved in apoptosis [91]. However, there is still no research on the relationship between the expression and function of miR-637 and DAPK3 in cancer. An in-depth study of the relationship between miR-637 and DAPK3 may broaden the understanding of the molecular mechanism of DAPK3 in cancer.

miRNAs are generally believed to localize to the cytoplasm to regulate translation [36]. However, emerging research has found that miRNAs can also exert transcellular regulatory roles in the form of exosomes. For example, cancer-associated fibroblasts regulate cellular metabolism in prostate and pancreatic cancers through miRNA-containing exosomes [92]. In addition, the upstream ceRNAs of miR-637 (circPSMA1 and circ_0000284) were upregulated in exosomes, thereby affecting the malignant phenotype of cells [36, 37]. This may be how miR-637 affects Jak/STAT3 and WNT signaling, but more data are still needed to support this hypothesis.

At present, the relationship between miR-637 in CRC and CHOL and some anticancer drugs has been studied. There are still a lot of deficiencies in the research on the role of miR-637 and different drugs, such as the role of miR-637 in other cancer treatment processes, etc. In future studies, changes in the expression of miR-637 during chemotherapy treatment in other cancers will be monitored to explore the role of miR-637 in cancer patient prognosis. Further research with an expanded sample size should be conducted on different cancer patients and different drug regimens, to better understand the relationship between the abnormal expression of miR-637 in cancer and the effect of drug treatment.

## Conclusions

This work provides a systematic overview of miR-637, points out the potential of miR-637 to become a hot spot in cancer research, and provides clues and directions for subsequent research on miR-637. In the future, it is necessary to further study the molecular mechanism of miR-637 and its impact on the efficacy of tumor therapeutic drugs, so as to lay a theoretical foundation for the clinical targeted therapy of miR-637 in tumors.

## Data Availability

Not applicable.

## Abbreviations

BRCA: Breast cancer
ccRCC: Clear cell renal cell carcinoma
ceRNA: Competing endogenous RNA
CHOL: Human cholangiocarcinoma
circRNA: Circular RNA
CML: Chronic myeloid leukemia
CRC: Colorectal cancer
CCa: Cervical cancer
DAPK3: Death associated protein kinase 3
DDP: Cisplatin
dFdC: Gemcitabine
DFS: Disease-free survival
EMT: Epithelial-mesenchymal transition
GBM: Glioblastoma multiforme
GC: Gastric cancer
HCC: Hepatocellular carcinoma
IM: Imatinib
LGG: Low-grade glioma
LIF: Leukemia inhibitory factor
lncRNA: Long non-coding RNA
miRNA: MicroRNA
MM: Multiple myeloma
mRNA: Messenger RNA
ncRNA: Non-coding RNA
NSCLC: Non-small cell lung cancer
OC: Ovarian cancer
OS: Overall survival
OSCC: Oral squamous cell carcinoma
OV: Ovarian cancer
OXA: Oxaliplatin
PCa: Prostate cancer
PDAC: Pancreatic ductal adenocarcinoma
PTC: Papillary thyroid carcinoma
RFS: Relapse-free survival
SaOS: Osteosarcoma
TC: Thyroid gland carcinoma
TNBC: Triple-negative breast cancer
TNM: Tumor node metastasis
